# Initial Evidence for the Efficacy of an Everyday Memory and Metacognitive Intervention

**DOI:** 10.1093/geroni/igaa054

**Published:** 2020-10-26

**Authors:** Ann Pearman, Emily Lustig, MacKenzie L Hughes, Christopher Hertzog

**Affiliations:** School of Psychology, Georgia Institute of Technology, Atlanta, USA

**Keywords:** Cognitive aging, Everyday functioning, Memory training

## Abstract

**Background and Objectives:**

The objective of this paper is to demonstrate the efficacy of an Everyday Memory and Metacognitive Intervention (EMMI) designed to improve everyday functioning of older adults. The EMMI emphasizes self-regulation as a behavioral approach to take priority over habitual behaviors that often impede everyday functioning.

**Research Design and Methods:**

This study used a quasi-experimental design (intervention vs waitlist control) to test whether the EMMI improved several aspects of everyday cognition. Thirty-three EMMI participants (*M*_age_ = 70.24) were compared to 20 control participants (*M*_age_ = 71.70 years). The 2 groups were compared on everyday memory failures and successes, measures of well-being, subjective memory, and a prospective memory task.

**Results:**

Participants who received the EMMI reported more memory successes and fewer memory failures over a 10-day measurement period postintervention. In addition, EMMI participants reported significantly higher life satisfaction and better subjective memory at posttest than the control group. Critically, the EMMI participants performed better on a laboratory contact prospective memory task.

**Discussion and Implications:**

The results from this study suggest that the EMMI is a promising approach that has potential to improve everyday memory functioning and perhaps help extend functional independence. Future studies will include randomized controlled trials as well as electronic measurement of memory incidents.

**Translational Significance:** This intervention uses a metacognitive self-regulatory approach to teach mindful goal pursuit, while fostering anticipation of cognitive challenges and the encouraging effective use of strategies (like spaced retrieval) and external aids (like lists and calendars). Participants’ memory self-efficacy and perceived control over memory improved, as did their everyday prospective memory, compared to a control group. These results indicate that it is possible to help older adults meet everyday cognitive challenges by training simple but effective approaches to manage current and future cognitive demands.

## Background and Objectives

Traditional memory training intervention studies with older adults work well in training memory strategies, but have had limited success in terms of transfer of training (e.g., Simons et al., 2016). Often training focuses on complicated mnemonics that can be burdensome to learn and difficult to actually use in real life (Brédart, 2019; Brooks et al., 1999; Derwinger et al., 2005; McKitrick et al., 1999). In particular, the evidence for improvement in everyday memory following memory strategy training is both scant and limited primarily to subjective memory (Hudes et al., 2019). We have argued that a different approach is needed to tackle the issue of improving the everyday memory experiences of older adults (Hertzog et al., 2020).

This paper reports initial evidence for the efficacy of an Everyday Memory and Metacognitive Intervention (EMMI). It focuses on improving older adults’ ability to achieve cognitively challenging everyday life tasks. It builds on other interventions that, although often focused on more traditional memory strategy training, have included everyday memory components in their interventions (e.g., Cohen-Mansfield et al., 2015; Troyer, 2001; West et al., 2008; Wiegand et al., 2013). Our approach is grounded in a metacognitive perspective and emphasizes self-regulation. We use a behavioral approach to supplement habitual behaviors that often impede everyday functioning (Dunlosky et al., 2011; Hertzog & Dunlosky, 2012). Although reliance on automated action routines has benefits, overreliance on habits can lead to costly everyday memory errors (Hertzog et al., 2019), especially when growing older leads to declines in the cognitive mechanisms that were critical for habits learned in young adulthood or midlife (Hertzog, 2008). The EMMI is based on several principles (Hertzog et al., 2020). First, people need to have a set of metacognitive skills to use in real-world situations. For instance, training spaced retrieval (Brédart, 2019; Camp, 2006; Walmsley & Fuqua, 2018; Wiegand et al., 2013) allows one to decide to learn someone’s name at a social gathering and access an effective tool for doing so. Second, we focus on creating a set of “habits-of-mind” (Stine-Morrow, 2007) that increase the likelihood of effective use of existing and newly trained memory aids. For instance, people often report using a calendar to support everyday memory but do not have a consistent habit of checking that calendar. Training people to create a new habit of consistent calendar referral can mitigate forgetting appointments (Chudoba et al., 2019).

A cornerstone of implementing a self-regulatory approach is fostering a form of self-reflection or mindfulness (Brown et al., 2016; Eyre et al., 2017; Levy et al., 2001), embodied in a “stop, think, plan, act” method that is designed to override reflexive, habitual behaviors (Wood & Rünger, 2016) by substituting goal-directed reflective thinking. For instance, leaving the house may involve a routine that is susceptible to forgetting (e.g., leaving behind important items), but reflecting on goals for the outing can create a new self-regulatory habit of checking to make sure all items needed are present (Burkard et al., 2014; West et al., 2009). We also evaluate and restructure (as needed) individuals’ memory beliefs to counteract negative beliefs that may inhibit engagement with our techniques. Finally, our intervention combines a standardized set of intervention components with a personalized programmatic approach to learning. While we teach basic everyday memory techniques in a classroom format, we also adapt to each participant’s stated and assessed challenges and goals as revealed by an intake interview and participant’s stated concerns.

The EMMI includes a unique three-stage training protocol. The first stage is an in-depth semistructured interview with each participant to identify each person’s current behaviors, habits, and daily challenges. During this interview, we encourage participants to both describe and start to evaluate their current ways of engaging with their everyday cognition. The information gained in these interviews is used for personalizing components of the intervention, including goal setting and shaping. The next stage is a group learning experience (GLE; see Table 1), which focuses on memory belief restructuring and trains basic memory skills and habits of mind for 3 days over the course of 1 week. Targeted skills include teaching and practicing several memory strategies known to be effective in everyday life, including spaced retrieval and implementation intentions. The new habits-of-mind encourage mindful reflecting on daily tasks and cultivating new habits of reviewing goals, plans, and actions during the course of the day. The first 2 days of the GLE have homework to be completed prior to the next session. The final stage of the EMMI is a behavioral shaping procedure, which is also a unique aspect of this program. This stage involves a 4-week shaping period of intensive individualized contact, primarily through phone calls with the research team, to monitor participants’ progress in mastering trained skills, analyze causes of memory failures, reinforce memory successes, and shape habit formation change.

**Table 1.** Summary of Group Learning Experience (GLE)

Topic	Content
Day 1	
Beliefs about memory	Restructuring memory beliefs to benefit performance
Intentional encoding	Discuss importance of intentional encoding and encourage participants to think about ways they do/don’t intentionally encode new information
Active noticing	Teach the mindful technique of being aware of one’s surroundings and experiences
Spaced retrieval	Explanation of spaced retrieval and its benefits
	Practice with learning the names of people in class
Homework	Memory belief restructuring in daily life
	New name learning for next class
Day 2	
Homework review	Review homework assignments
	Practice class and research team names
Self-testing	Explanation of self-testing, how it relates to intentional encoding, and ideas about how to use it in daily life
Habits and routines	Discussion of the pros and cons of habits and routines
	Identification of personal habits/routines that may interfere with rather than promote functioning
Implementation intentions	Explanation about setting intentions to help with prospective memory tasks
Stop, Think, Plan Act (STPA)	Introduction of STPA and how it can be used in daily life to enhance memory
	Identification of possible personal uses for STPA
Homework	Attend to and identify personal habits and routines
	Practice using STPA in daily life
	Self-testing practice
Day 3	
Homework review	Review homework assignments
	Practice class and research team names
Review of STPA	Review STPA
External aids	Discuss “optimal” calendar and list use as well as medication adherence
Mindfulness	Introduction to mindfulness and how it relates to managing everyday memory demands
Homework	Handing out and explanation of daily diaries
	Explanation of shaping period that will occur next in study

We used a quasi-experimental design (Shadish et al., 2002) for this study, comparing a group of EMMI-trained older adults to a waitlist control group. We tested several a priori hypotheses:

(1) Trainees would show greater improvements from pretest to posttest on subjective memory beliefs compared to the control group.
(2) Trainees would show declines in stress and improvements in life satisfaction compared to the control group.
(3) Trainees would have fewer reported memory failures and more memory successes compared to the control group.
(4) Trainees would perform better on a laboratory contact task assessing everyday prospective memory compared to the control group.

## Research Design and Methods

### Participants

Participants aged 65 and older were recruited from the greater Atlanta area for the intervention study. Recruitment sources included the Georgia Tech Silver Jackets, a social organization for retired staff, faculty, and their spouses; the Adult Cognition Lab participant database, a long-standing list of older adult research volunteers; and advertisements in a local senior magazine and a doctor’s office. In addition, we did a large postcard mailing using a purchased ZIP code-based mailing address list for the greater Atlanta area.

**Figure 1.**
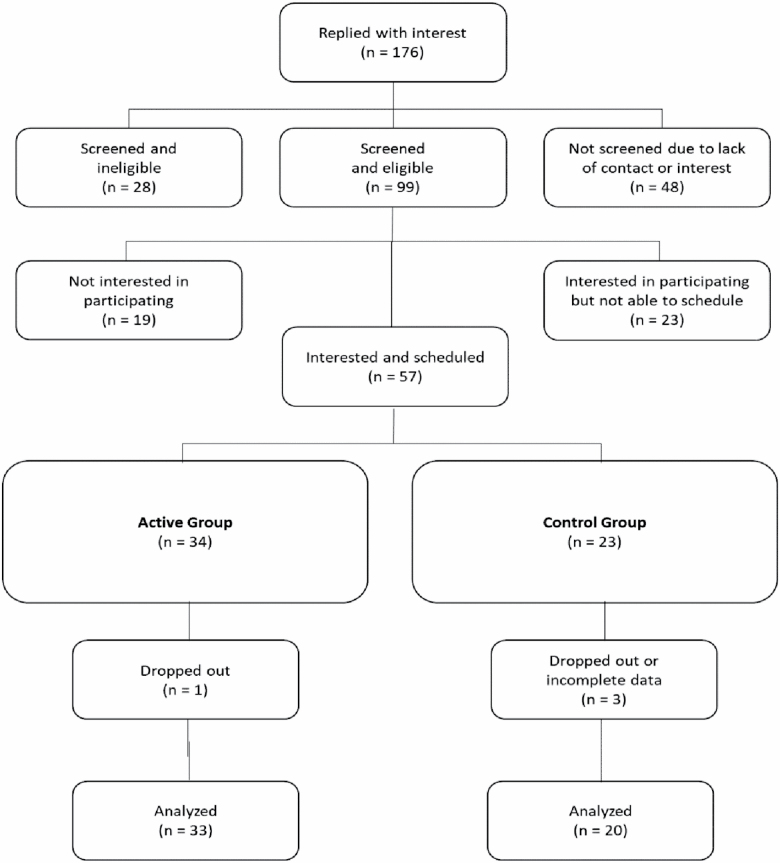
Flowchart of participant recruitment.

Potential volunteers called the laboratory phone number and were given detailed information about the study. If they continued to be interested, they were screened for eligibility. The following inclusion criteria were applied: age 65 or above, absence of a dementia diagnosis or recent stroke, scores above 17 on the Montreal Cognitive Assessment (MoCA; Nasreddine et al., 2005), and a willingness/ability to attend group training sessions held on the Georgia Tech campus. Once eligibility was determined, participants were assigned to either an EMMI or control group (see Figure 1 for participant flow). We chose 17 on the MoCA to allow for participants who might have mild cognitive impairment range as we believe our intervention is applicable to a wide range of capabilities.

### Design and Procedure

The design was quasi-experimental in nature. Given the novel intervention approach, we started data collection with the EMMI group to enable an evaluation of acceptance and compliance to intervention procedures, and then phased in collection of the control group. Both groups were drawn from the same sampling frame over a period of 15 months (December 2018–March 2020). Control group participants were enrolled after identical recruitment procedures, except that they were told they were in a control group and would be offered the intervention after the posttest. The major difference in study protocol between the two groups was that control participants were enrolled later in the study than intervention participants.

The intervention was separated into the three parts: intake interviews, group training sessions administered at Georgia Tech, and a 4-week shaping period.

The aforementioned semistructured intake interviews were used to identify how people organized their daily lives and the procedures they typically used to manage everyday memory. The interview script, which was based on Hertzog et al. (2019), specifically queried multiple potential memory strategies with follow-up questions to identify any concerns or issues experienced by the participant. This interview also is used for individualization of the intervention components during group training and shaping.

The EMMI group training sessions were held on three weekdays (e.g., Monday, Wednesday, Friday) for a 3-hr period each day. EMMI participants were trained in small groups typically ranging from three to five persons. Each session consisted of a PowerPoint-mediated presentation, including frequent group discussion and sharing, organized activities, and breakout sessions for one-on-one training and recommendations from research staff. Participants were given homework assignments to reinforce trained procedures and reported back to the group during the next session on their experiences. Table 1 shows the topics covered in every session.

The shaping period involved daily diary completion along with intensive telephone contact from staff with trainees to review contents of the diary and to discuss everyday memory experiences (e.g., reported memory successes and failures [termed “blips” to avoid negative connotations surrounding “failure”], any difficulty using trained memory procedures, goal setting for future use, and other memory-related topics of interest to participants). Frequent contact was made initially, tapering to less frequent contact over the 4-week period (e.g., week 1 had four calls and week 4 had one call). Participants received up to 10 scheduled calls with each one lasting approximately 15 min. The script for the shaping calls can be found in Supplementary Material. 

The design involved six components: (1) intake interview; (2) pretest data collection; (3) group intervention sessions (EMMI participants only); (4) shaping period with concurrent daily diary recordings of everyday memory experiences (only EMMI participants received shaping, as described below); (5) posttest data collection; and (6) group discussion of intervention and debrief. Control participants did not engage in the group intervention sessions (3) or the shaping component of (4), but did complete daily diaries for up to 37 consecutive days. They were given the intervention, if desired, between phases (5) and (6).

Fifty-seven participants, 21 males and 36 females, aged 70.79 years (*SD* = 3.84, range 65–83) were initially recruited into this study. One intervention and three control participants dropped out of the study after completing the intake interview and pretest. One control participant was excluded from the analyses due to completing less than 50% of the daily diaries in the last 10-day assessment period. Exclusion due to compliance rates of less than 50% are standard criteria for this type of study. The final sample included data from 53 participants with a mean age of 70.79 years (*SD* = 3.91): 33 intervention participants (*M*_age_ = 70.24) and 20 control participants (*M*_age_ = 71.70 years). Characteristics of the final sample are shown in Table 2.

**Table 2.** Sample Characteristics by Group

Variable	Intervention (*n* = 33) *M* (*SD*) or *N* (%)	Control (*n* = 20) *M* (*SD*) or *N* (%)
Age	70.2 (3.2)	71.7 (4.8%)
Sex^a^		
Female	17 (51.5%)	17 (85.0%)
Male	16 (48.5%)	3 (15.0%)
Hispanic/Latino	2 (6.1%)	0 (0.0%)
Non-Hispanic/Latino	31 (93.9%)	17 (85.0%)
Do not wish to answer	0 (0.0%)	2 (10.0%)
Race		
African American	13 (39.4%)	7 (35.0%)
Caucasian	19 (57.6%)	12 (60.0%)
Other	1 (3.0%)	1 (5.0%)
Education		
Some high school	1 (3.0%)	2 (10.0%)
High school graduate or equivalent	3 (9.1%)	3 (15.0%)
2-year college or vocational school degree	1 (3.0%)	0 (0.0%)
Some college (no degree)	2 (6.1%)	2 (10.0%)
Bachelor’s degree	10 (30.3%)	5 (25.0%)
Some graduate school (no degree)	2 (6.1%)	4 (20.0%)
Master’s degree	9 (27.3%)	2 (10.0%)
JD, MD, or PhD	5 (15.2%)	2 (10.0%)
Subjective health	3.88 (0.93)	3.95 (0.83)
MoCA	25.58 (3.21)	25.10 (2.69)
RBANS		
Immediate List Memory	27.00 (4.68)	26.80 (3.41)
Immediate Story Memory	15.61 (4.11)	16.35 (3.94)
Delayed List Memory	6.33 (2.23)	5.80 (2.48)
Delayed Story Memory	8.70 (2.22)	8.95 (2.26)

*Notes*: MoCA = Montreal Cognitive Assessment (Nasreddine et al., 2005); RBANS = Repeatable Battery for the Assessment of Neuropsychological Status (Randolph, 1998).

^a^The only significant differences between the groups at baseline was sex with the control group having significantly more women, χ ^2^(1) = 6.07, *p* < .05.

The study was approved by the Georgia Institute of Technology Institutional Review Board. No incidents or protocol deviations occurred during the study.

### Measures

#### Pretest/posttest measures

##### Memory beliefs

The Personal Beliefs about Memory Inventory (PBMI) measured subjective memory beliefs (Lineweaver & Hertzog, 1998). It consists of 57 items asking participants to rate their own memory on visual analog rating scales. The derived scales are: Global Memory rating (1 item), Global Memory rating relative to people of all ages (1 item), Global Memory rating relative to same-age peers (1 item), Specific Memory Self-Efficacy (MSE), Retrospective Change, Prospective Change, Current Control, Prospective Control, and Future Control. Higher scores represent better subjective memory or less decline. For purposes of this study, we focus on ratings of current memory and control. Because the sample included participants over the age of 75 years, we omitted four items from the Prospective Change variable that were specifically directed to a future age of 75 years (Cronbach’s alpha = .97).

##### Well-being

The 10-item Perceived Stress Scale (PSS; Cohen & Williamson, 1988) was used to measure perceptions of stress. The scale includes questions about levels of experienced stress over the past week (e.g., In the last week, how often have you found that you could not cope with all the things that you had to do?). Participants answer each item on a 0 (*never*) to 4 (*very often*) scale. Items were coded or reverse-recoded such that higher scores indicate more stress. A summary score is calculated for each participant (Cronbach’s alpha = .80).

Participants’ life satisfaction was assessed with the five-item Satisfaction with Life Scale (Diener et al., 1985). Participants rated items (e.g., the conditions of my life are excellent) on a seven-point scale from 1 (*strongly disagree*) to 7 (*strongly agree*). A sum score was calculated with higher scores representing higher life satisfaction (Cronbach’s alpha = .83).

##### Objective memory

The immediate and delayed memory scales from the Repeatable Battery for the Assessment of Neuropsychological Status (RBANS; Randolph, 1998) were used to measure episodic memory. The RBANS is designed to measure cognitive decline or improvement. Four alternate forms are available for each subtest to avoid re-administration of the same materials. Participants were administered the Story Memory and List Learning subtests (both immediate and delayed) at both pretest and posttest. Forms were counterbalanced across participants.

#### Other outcome measures

##### Daily diary

Participants were administered a short online daily diary for 5 weeks that assessed aspects of daily experience associated with everyday memory. Diary templates can be found in the Supplementary Material. Briefly, individuals were asked each night to describe any memory successes and failures they experienced, to check any strategies they had used to support their memory during the day, and to complete rating scales about daily stress and affect. For EMMI participants, daily diaries were used for training purposes during the shaping period and checked by project staff to monitor compliance. A few (i.e., four EMMI and one control) participants did not have the technical capability of using the online diary and were instead allowed to complete hard copies of the daily diaries. For the purpose of comparable between-group comparisons, we used the last 10 diary days (post-shaping for the EMMI group) to extract relevant outcomes. Both groups completed these last daily diaries without further contact or interaction with the project staff. The groups completed a similar number of nightly diary reports over the final 10 days of diaries: EMMI *M* = 9.30 diaries (*SD* = 1.05, range = 7–10), control group *M* = 8.80 diaries (*SD* = 1.32, range = 5–10).

There were several key outcome variables derived from the diaries. First, participants answered the question “Did you experience any problems remembering something today?” with a yes or no response. This variable was aggregated across the last 10 days of diaries and adjusted for missing diaries. Next, we coded for both memory failures and successes, using information qualitatively recorded anywhere in the diary that spoke to a specific memory failure or success of some kind. Two research assistants independently coded these variables, then resolved any disagreements by consensus.

Finally, to examine use of trained strategies by EMMI participants, we harvested information from the strategy checklist about everyday strategies that were used, creating dichotomous (yes/no) variables about whether a memory strategy had been used on a given day. We augmented the strategy variables if information in any other part of the diary indicated use of a strategy not checked in the checklist. Such incidents were relatively infrequent (4.31% of the total strategies were reported outside of the checklist). One research assistant examined all diaries and identified strategies listed outside of the strategy checklist; another research member reviewed the additional strategies for consensus. All three variables were scaled as the number of reported incidents divided by the number of provided daily reports to adjust for missing data. For EMMI participants, the strategies variable was aggregated into four categories: (1) habits and routines (i.e., follow a routine); (2) internal aids (i.e., intentional encoding, retrieval practice, active noticing); (3) external aids (i.e., had someone else remind you, smartphone alarm, lists, reminder notes, appointment book/calendar, leave things in familiar places); and (4) self-regulatory strategies (i.e., mindfulness, implementation intentions, self-testing, Stop, Think, Plan Act). The control group endorsed a reduced set of strategies that did not include trained strategy terminology explicitly used with the EMMI group.

##### Prospective memory task

Participants were given five times/dates and were instructed to contact our laboratory (by call, e-mail, or text). The instructions were provided to EMMI participants at the end of the second group training session and to control participants during their intake interview session. All participants were told these contacts would be recorded as a way of measuring their ability to complete a task on schedule, and that they could use any means of remembering to complete the contacts. After the schedule was provided and instructions on how to do the task were reviewed, the task was not mentioned again thereafter by the research team. Exact date and time of contact were recorded by the research team. Two scores were then derived from this information. The first was the number of contacts (0–5) successfully completed in a time window 15 min before or 15 min after the designated time. Participants received a score of 1 for completing the task within the 15 min before or after their scheduled time or received a score of 0. The second measure scaled the difference between scheduled versus actual completed contacts. The derived index was the median absolute deviation of actual call time from scheduled call time for calls completed within a window of 60 min before the scheduled call time to 120 min after the scheduled call time.

### Analyses

χ ^2^ tests and *t* tests were used to evaluate any potential baseline (pretest) differences between the groups. For variables that were administered at both pretest and posttest (PBMI, Life Satisfaction, PSS, and RBANS), we used a repeated measures general linear model (GLM) to evaluate change from pretest to posttest and differential change between the two groups. For all other measures (daily diary reports and prospective memory), we conducted GLMs to evaluate postintervention differences between groups. Given the quasi-experimental design, we evaluated these intervention effects while controlling on the covariates of age, sex, education, race (Caucasian vs Other), MoCA for intervention versus control groups as Cohen’s *d* and an adjusted Cohen’s *d* (controlling on covariates), using the square root of residual mean square error to scale the mean differences.

## Results

### Pretest Group Comparisons

We examined the pretest data to evaluate any differences between the intervention and control groups prior to the intervention (see Table 2). The control group was disproportionately female (χ ^2^(1) = 6.07, *p* < .05). There were no group differences in age, race, years of education, self-rated overall health, or self-rated overall memory. The intervention and control group did not differ in pretest MoCA (*t*(51) = 0.55, *p* = .58) or RBANS tests, including Immediate List Memory (*t*(51) = 0.17, *p* = .90), Immediate Story Memory (*t*(51) = −0.65, *p* = .52), Delayed List Memory (*t*(51) = 0.81, *p* = .42), and Delayed Story Memory (*t*(51) = −0.40, *p* = .69). The two groups also did not differ at baseline on the variables measured both before and after the intervention: Life Satisfaction, PBMI, and PSS (see Table 3 for details).

**Table 3.** Pretest to Posttest Change by Group

	Intervention *M* (*SD*)				Control *M* (*SD*)			
Measure	Pre	Post	Cohen’s *d*^*a*^	Cohen’s *d*^*b*^	Pre	Post	Cohen’s *d*^*a*^	Cohen’s *d*^*b*^
PBMI Global Memory	0.62 (0.20)	0.73 (0.16)	0.55	0.45	0.65 (0.20)	0.63 (0.20)	−0.10	−0.08
Specific MSE	0.65 (0.14)	0.71 (0.13)	0.43	0.35	0.69 (0.13)	0.66 (0.14)	−0.23	−0.29
Retrospective Change	0.38 (0.15)	0.47 (0.18)	0.60	0.45	0.41 (0.13)	0.42 (0.12)	0.08	0.00
Prospective Change	0.39 (0.14)	0.44 (0.14)	0.36	0.23	0.42 (0.16)	0.41 (0.14)	−0.06	0.00
Relative to People of All Ages	0.51 (0.20)	0.60 (0.22)	0.45	0.23	0.53 (0.18)	0.54 (0.21)	0.06	0.15
Relative to Same-Age Peers	0.61 (0.16)	0.68 (0.19)	0.44	0.22	0.45 (0.16)	0.43 (0.12)	−0.13	0.09
Current Control	0.80 (0.14)	0.86 (0.09)	0.43	0.49	0.53 (0.18)	0.54 (0.21)	0.06	−0.28
Prospective Control	0.73 (0.17)	0.79 (0.14)	0.35	0.35	0.78 (0.16)	0.75 (0.14)	−0.19	−0.20
Future Control	0.66 (0.18)	0.76 (0.12)	0.56	0.60	0.76 (0.18)	0.70 (0.17)	−0.33	−0.45
Life Satisfaction Scale	25.55 (5.87)	27.36 (5.30)	0.31	0.18	23.75 (6.26)	22.75 (6.85)	−0.16	−0.06
Perceived Stress Scale	10.12 (5.96)	8.09 (5.61)	−0.34	−0.23	12.45 (7.00)	12.80 (7.11)	0.05	0.00
RBANS								
Immediate List Memory	27.00 (4.68)	29.36 (4.16)	0.50	0.44	26.80 (3.41)	28.85 (4.92)	0.60	0.63
Immediate Story Memory	15.61 (4.11)	16.03 (3.63)	0.10	0.12	16.35 (3.94)	15.95 (4.72)	−0.10	−0.11
Delayed List Memory	6.33 (2.23)	7.61 (1.73)	0.57	0.56	5.80 (2.48)	6.90 (1.68)	0.44	0.48
Delayed Story Memory	8.70 (2.22)	9.15 (2.24)	0.20	0.18	8.95 (2.26)	8.85 (2.39)	−0.04	0.06

*Notes*: MSE = Memory Self-Efficacy; PBMI = Personal Beliefs about Memory Inventory (Lineweaver & Hertzog, 1998); RBANS = Repeatable Battery for the Assessment of Neuropsychological Status (Randolph, 1998).

^a^Cohen’s *d* for pretest–posttest change, not adjusted for covariates, is scaled as (*M*_Posttest_ – *M*_Pretest_) / *SD*_Pretest_ separately in each group. ^b^Cohen’s *d* for pretest–posttest change, adjustment for covariates, is scaled as (LSM_Posttest_ – LSM_Pretest_) (MSE^−0.5^) separately in each group, where LSM is the fitted least squares mean for each group and MSE is the mean square error from the general linear model. Covariates include age, sex, health, education, race, MoCA, and RBANS composite (except in RBANS analyses).

### Intervention Effects on Subjective Memory, Life Satisfaction, and Stress

#### Personal memory beliefs

We hypothesized that the intervention would lead to increases in memory self-efficacy and perceived memory control. Given that the PBMI was administered at pretest and posttest, we evaluated intervention effects in a Group (Intervention vs Control) × Time (Pretest–Posttest) repeated measures GLM including covariates and Covariate × Time interactions. Relevant means and standard deviations are reported in Table 3. We detected statistically reliable interactions on most PBMI subscales. The interaction for Global Memory ability (*F*(1, 44) = 7.78, *p* = .008, η _p_^2^ = .15) reflected the intervention group reporting a significant increase in Global Memory ability from pretest to posttest (*F*(1, 44) = 16.37, *p* < .001, η _p_^2^ = .27), but no such effect was seen in controls (*F <* 1). No significant Group × Time interactions were detected when memory ability was rated either relative to same-age peers or relative to people of all ages, *F*s < 1.

For the critical PBMI Self-Efficacy scale, there was a robust Group × Time interaction, *F*(1, 44) = 12.94, *p* < .001, η _p_^2^ = .23. The intervention group’s MSE increased from pretest to posttest, *F*(1, 44) = 14.45, *p* < .001, η _p_^2^ = .25, whereas the control group experienced a nonsignificant decrease, *F*(1, 44) = 3.53, *p* = .07, η _p_^2^ = .07.

On the PBMI Retrospective Change scale, the significant Group × Time interaction, *F*(1, 44) = 5.25, *p* = .027, η _p_^2^ = .11 reflected the same pattern. The intervention group reported a significant increase (indicating reduced perceptions of memory decline) from pretest to posttest, *F*(1, 44) = 15.33, *p* < .001, η _p_^2^ = .26, whereas the perceived change of the control group did not differ from pretest to posttest, *F* < 1. There was no significant pretest–posttest difference in anticipated future changes, as measured by the PBMI Prospective Change scale, *F* < 1.

Perceived control over memory, as captured by PBMI Current Control, generated a reliable Group × Time interaction, *F*(1, 44) = 12.94, *p* < .001, η _p_^2^ = .23. The intervention group reported a significant increase in control from pretest to posttest, *F*(1, 44) = 15.08, *p* < .001, η _p_^2^ = .26, whereas the control group did not, *F*(1, 44) = 3.29, *p* = .08, η _p_^2^ = .07. A similar interaction was found for PBMI Prospective Control, *F*(1, 44) = 9.77, *p* = .003, η _p_^2^ = .18, assessing beliefs in control over future memory demands. The intervention group increased perceived control over future memory demands, *F*(1, 44) = 11.31, *p* = .002, η _p_^2^ = .20, unlike the control group, who showed a nonsignificant decrease in perceived control (*F*(1, 44) = 2.52, *p* = .12, η _p_^2^ = .05. For PBMI Future Control, the reliable Group × Time interaction, *F*(1, 44) = 22.94, *p* < .001, η _p_^2^ = .34 indicated differential improvement in beliefs about ability to control memory in the future, with the intervention group reported a significant increase, *F*(1, 44) = 23.47, *p* < .001, η _p_^2^ = .35, whereas the control group’s belief in future control reliably decreased, *F*(1, 44) = 7.09, *p* = .011, η _p_^2^ = .14.

#### Life satisfaction

Although there was a significant Group × Time interaction on the Satisfaction with Life Scale without control on covariates, this effect missed significance when partialled for the covariates, *F*(1, 44) = 3.21, *p* = .08, η _p_^2^ = .07. Nevertheless, EMMI participants reported reliable increases in life satisfaction from pretest to posttest in the model including covariates, *F*(1, 44) = 5.34, *p* = .026, η _p_^2^ = .11, whereas there was little change in control participants, *F* < 1.

#### Perceived stress

A reliable main effect of Group on the PSS was eliminated when controlling on the covariates. More critically, there was no Group × Time interaction, *F*(1, 44) = 1.18, *p* = .28, η _p_^2^ = .03.

### Intervention Effects on Everyday Memory

Table 4 reports mean differences between the EMMI intervention group and the control group on posttest-only outcome measures that were analyzed in a GLM with the same set of covariates. We report effect size statistics both with and without adjustment for covariates.

**Table 4.** Laboratory Contact Task Performance and Daily Diary Reports by the Intervention and Control Groups

Task	Intervention *M* (*SD*)	Control *M* (*SD*)	Cohen’s *d*^a^	Adjusted Cohen’s *d*^b^
Telephone call-in task				
Number of calls completed	3.24 (1.50)	1.90 (1.37)	0.93	0.79
Median absolute deviation	2.69 (5.90)	13.06 (16.90)	−0.82	−0.88
Daily memory problems	0.24 (0.27)	0.37 (0.22)	−0.53	−0.48
Daily memory successes	0.62 (0.32)	0.46 (0.36)	0.47	0.34
Daily memory blips	0.42 (0.45)	0.56 (0.31)	−0.36	−0.40
Daily memory strategies				
Follow a routine	0.31 (0.31)	0.22 (0.33)	0.28	0.25
External	0.23 (0.18)	0.16 (0.16)	0.42	0.42
Self-regulatory^c^	0.21 (0.11)	—	—	—
Internal^c^	0.14 (0.12)	—	—	—

*Note*: ^a^Cohen’s *d* without adjustment for covariates. Scaled so that a positive score indicates greater means for Intervention group; *d* = (*M*_Intervention_ − *M*_Control_) (MSE^−0.5^), where MSE is the mean square error from the general linear model (GLM). ^b^Cohen’s *d* with adjustment for covariates (age, sex, health, education, race, Montreal Cognitive Assessment, and Repeatable Battery for the Assessment of Neuropsychological Status composite). Scaled so that a positive score indicates higher performance by Intervention group; *d* = (LSM_Intervention_ − LSM_Control_) (MSE^−0.5^), where LSM is the covariate-adjusted least squares fitted mean for that group and MSE is the mean square error from GLM with covariates. ^c^Strategies reported in Intervention group daily diaries only.

#### Daily diary reports

##### Reported memory failures and successes

Results for these variables differed depending on whether covariates were used in the analysis. As can be seen in Table 4, the intervention group reported significantly fewer days with memory problems compared to the control group, one-tailed *p <*.05. Per the request of a reviewer, we reran the analyses including the control participant who had only 10% completion of their daily diaries. When this participant was included in the analyses, group differences between days with memory problems remained significant [*M*_EMMI_ = 0.24 (*SD* = 0.27) vs *M*_control_ = 0.40 (*SD* = 0.26), *t*(52) = −2.19, one-tailed *p* < .05, Cohen’s *d* = 0.60], memory successes per day no longer reached significance [*M*_EMMI_ = 0.62 (*SD* = 0.32) vs *M*_control_ = 0.48 (*SD* = 0.37), *t*(52) = 1.41, one-tailed *p* = 0.09, Cohen’s *d* = 0.40)], and memory failures per day reached significance [*M*_EMMI_ = 0.42 (*SD* = 0.45) vs *M*_control_ = 0.63 (*SD* = 0.44), *t*(52) = −1.70, one-tailed *p* < .05, Cohen’s *d* = 0.47)]. The intervention group also reported a significantly greater number of memory successes per day compared to the control group, *t*(51) = 1.68, one-tailed *p* <.05. For the coded memory failures, the intervention group described fewer memory failures per day compared to the control group’s memory failures per day, but group differences did not reach statistical significance, *t*(49.83) = −1.36, one-tailed *p =* .09.

However, many of these effects did not survive adjustment for covariates, with nonsignificant group differences for the three outcome variables: memory problems, *F*(1, 44) = 2.31, *p* = .14; memory successes, *F*(1, 44) = 1.23, *p* = .27; and memory failures, *F*(1, 44) = 0.24, *p* = .63. For memory failures, effects of the covariates MoCA, education, and sex were reliable, suggesting that these variables might have generated the group differences. However, inspection of Table 4 shows that inclusion of covariates did not materially reduce effect sizes on adjusted marginal means, which suggests that failure to detect effects for memory problems and memory successes might have been due to reduced degrees of freedom in the residual term (and hence, lower statistical power).

##### Use of trained memory strategies by EMMI participants

The EMMI group indicated which memory-supportive strategies they used each day in the diaries. Strategies were categorized into the following subtypes: Habits and Routines, External, Internal, and Self-Regulatory. Habits and Routines was endorsed most frequently (*M* = 0.31, *SD* = 0.31) followed by External Strategies (*M* = 0.23, *SD* = 0.18), Self-Regulatory Strategies (*M* = 0.21, *SD* = 0.11), and finally Internal Strategies (*M* = 0.14, *SD* = 0.12). The levels of self-regulatory strategy use in particular indicated that EMMI participants were using trained strategies after the intervention, given that these behaviors appear to be uncommon in older adults (Hertzog et al., 2010).

Because many of these measured strategies used terminology from the intervention GLEs (e.g., Stop, Think, Plan Act), we only asked the control group about strategies that had a clear meaning outside of the intervention, including External Strategies (e.g., used a calendar) and Habits or Routines (e.g., followed a routine). External aid use is commonly reported by older adults, and the intervention sought to optimize external aid use, building on that base. We therefore did not expect group differences in frequency of using these strategies. As can be seen in Table 4, EMMI participants reported more frequent external aid use in the daily diaries than controls, although the difference did not achieve statistical significance, *F*(1, 44) = 1.77, *p* = .19, η _p_^2^ = .04. The difference in sample means for Habits and Routines was not reliable, *F* < 1.

### Prospective Memory



##### Prospective memory laboratory contact task

The EMMI group outperformed the control group on the laboratory contact task, successfully making more calls within the designated completion window (see Table 4), *F*(1, 44) = 6.19, *p* = .017, η _p_^2^ = .12. EMMI participants also completed their calls closer to the scheduled call time, as measured by the absolute deviation between scheduled time and actual call time, *F*(1, 38) = 6.66, *p* = .014, η _p_^2^ = .15. As can be seen in Table 4, the standardized effect sizes were large in magnitude.

### Intervention Effects on Episodic Memory

We did not expect our everyday memory intervention to affect traditional memory test scores. Table 3 reports the pretest and posttest means on the RBANS subscales. There were no Group × Time interaction effects on the four tests, Immediate and Delayed List Memory and Immediate and Delayed Story Memory (all *F*s < 1).

## Discussion and Implications

The overall message of this paper is one of initial success and future promise for the EMMI, with results indicating effectiveness in improving the everyday memory of the participants. As with other intervention studies with an everyday training component, we detected differential increases in subjective memory after EMMI (e.g., Troyer, 2001; Wiegand et al., 2013; see Hudes et al. 2019). However, we also produced evidence of changes in everyday memory behavior as well. Intervention participants made more scheduled laboratory contacts within the allotted time window, also making those contacts closer to the scheduled time than control participants. The intervention produced large effect sizes for these variables based on Cohen’s benchmarks (Cohen, 1988). The effect on the laboratory contact task was particularly important, as it represents a method for assessing everyday prospective memory that is widely used (e.g., Henry et al., 2004; Troyer, 2001) and not subject to self-report validity concerns.

In the diaries, EMMI participants reported significantly more memory successes, a lower frequency of reported everyday memory problems, and a trend for a lower level of specific reported memory failures in nightly diaries collected over the 10-day assessment period, compared to the control group. However, these effects were not statistically reliable when covariates were included in the analysis. We are nevertheless encouraged to use these measures in a future randomized controlled trial with a sufficient sample size to have power to detect small to moderate effects.

We view these outcomes as an important departure from more traditional memory training studies that typically measure memory performance outcomes but not everyday memory itself. It is therefore notable that the intervention did not differentially improve performance on the RBANS memory test, underscoring that the EMMI training improved everyday memory behaviors rather than strategies that benefit memory test performance.

Regarding the intervention effects on subjective memory (Hertzog & Pearman, 2014), this outcome is important. People believing that their everyday memory has improved is the kind of impact valued by older adults. In addition, reported life satisfaction changed differentially for EMMI participants, hinting that subjective memory improvement may have had benefits for general psychological well-being.

In sum, then, these results are highly encouraging for the potential for this approach to modify functional competence and independence of older adults despite age-related memory changes.

### Limitations

There are several limitations to this study. The nonrandom assignment of persons to groups and sampling issues led to a gender difference in composition of the EMMI group and control group. Another limitation is that the retrospective self-reports in nightly diaries can also be subject to memory failures (i.e., forgetting about forgetting), especially if these incidents are more of a nuisance than a major problem for everyday goal pursuit. We have developed a SmartPhone app that will allow our future participants to report memory incidents when they happen which will, hopefully, mitigate this limitation. Another limitation is that our sample size was relatively small. However, our hypothesized effects on the primary outcome measures were generally large enough to be detected by this design. Given the small sample size and the lack of a randomized active control condition (see Karbach & Verhaeghen, 2014; Simons et al., 2016), we cannot know how much of the observed effect is due to the social nature of the group training sessions. We aimed to establish personal rapport and comity in these sessions, and people had a chance to listen to each other’s experiences and reactions. The active participants had considerably more interaction with and support from the research team. As noted in Future Directions, our next study includes an active control group that will receive an equal amount of interaction with the research team. Finally, we were unable to include a long-term follow-up component of the intervention to test the sustainability of the effects.

### Future Directions

The results of the current study suggest that practitioners and researchers who are interested in helping older adults with everyday cognitive challenges might be well-served to use a metacognitive perspective that focuses on mindful memory practices and an assortment of easy-to-use memory techniques. This study is an important step in the process of transforming memory training from more traditional mnemonic programs to more everyday-friendly programs that work with older adults’ existing strengths and routines. We detail below our team’s next steps in this programmatic research and hope that other researchers will take up this push toward maximizing independence and everyday functioning as a goal of future work.

Our next step is to conduct a true randomized controlled trial with an active control group. We are in the process of running an intervention to contrast directly EMMI with a traditional memory strategy intervention control group while equating conditions for levels of interactions, training-gain expectations, and group training experiences (NCT: 04088136). We hypothesize that the EMMI will differentially improve everyday memory but not memory test performance. Conversely, we hypothesize that the memory strategy training group will improve on memory test performance but not everyday memory. This empirical dissociation would underscore the difference between processes that successfully train objective memory test performance versus those that impact everyday memory.

We conclude that this new intervention approach has potential to help older adults improve their everyday memory functioning. We believe it could also help older adults maintain functional independence even in the face of cognitive aging.

## Supplementary Material

igaa054_suppl_Supplementary_Materials_S1

igaa054_suppl_Supplementary_Materials_S2
