# Supplementary material for: Initial Evidence for the Efficacy of an Everyday Memory and Metacognitive Intervention
Source: Innov Aging. 2020 Oct 26;4(6):igaa054. doi: 10.1093/geroni/igaa054 (PMC7729280; doi:10.1093/geroni/igaa054)
Supplement: igaa054_suppl_Supplementary_Materials_S1 [file igaa054_suppl_supplementary_materials_s1.docx]

**Supplementary Material: DAILY DIARY**

Please list today’s major events (e.g., appointments, errands, social events):

On the whole, please rate today in terms of your feelings, thoughts, and actions:

|  | Strongly disagree | Disagree | Neutral | Agree | Strongly agree |
| --- | --- | --- | --- | --- | --- |
| I planned today’s activities before the start of the day |  |  |  |  |  |
| I achieved my goals today |  |  |  |  |  |
| I met unexpected obstacles or challenges today |  |  |  |  |  |
| I was able to adapt my plans as needed today |  |  |  |  |  |
| I experienced stress today |  |  |  |  |  |
| I experienced a memory challenge today |  |  |  |  |  |
| Overall, my memory today was very good. |  |  |  |  |  |

Rate your mood today:

|  | Very slightly or not at all | A little | Moderately | Quite a bit | Extremely |
| --- | --- | --- | --- | --- | --- |
| Determined |  |  |  |  |  |
| Attentive |  |  |  |  |  |
| Alert |  |  |  |  |  |
| Inspired |  |  |  |  |  |
| Active |  |  |  |  |  |
| Afraid |  |  |  |  |  |
| Nervous |  |  |  |  |  |
| Upset |  |  |  |  |  |
| Ashamed |  |  |  |  |  |
| Hostile |  |  |  |  |  |
| Happy |  |  |  |  |  |
| Sad or blue |  |  |  |  |  |
| Joyful |  |  |  |  |  |
| Angry |  |  |  |  |  |
| Frustrated |  |  |  |  |  |

*Did you experience any successes remembering something using the techniques we taught you? Yes No

Please describe these successes with a sentence or two.

Did you use any of the following aids to help you remember something today? (check all that apply)

| - Lists - Reminder notes - Appointment book/calendar - Had someone else remind you - Leave things in familiar places - Smartphone alarm - Follow a routine - Retrace your steps - Stop/Think/Plan/Act* | - Active noticing* - Self-testing* - Spaced retrieval* - Implementation Intentions* - Mindfulness* - Intentional Encoding* - None of the above - Other: |
| --- | --- |

*Did you experience any problems remembering something today? Yes No

Did you forget to do any of the following tasks today? We will ask you to tell us more about it on the following section (check all that apply)

- An errand/chore
- Take medicine
- Attend a meeting/appointment
- Make a phone call
- Why you entered a room
- Bring something with you
- None of the above
- Other, explain:

Did you have difficulties doing any of the following things today? We will ask you to tell us more about it on the following section (check all that apply)

- Keeping track of what you were doing
- Bringing a word to mind
- Being organized
- Making decisions
- Thinking quickly
- Thinking clearly
- Finding your way around
- Completing a task
- None of the above
- Other, explain:

Now we’d like to learn more about your experiences with remembering today, focusing on any difficulties you may have experienced. In the following pages please describe up to 3 events where you forgot something. We will ask you to tell us more about it, how important it was, and what you experienced at the time. Any type of memory blip could be reported, from a relatively major event to a minor nuisance. You can tell us about up to 3 events each day.

*Please describe any specific memory problems (up to 3) today with a sentence or two:

Please answer these questions about each memory problem:

- 1. Was this incident due to a disruption in your routine? Yes No
  2. How stressful was this incident for you? Not at all Mildly Moderately Extremely
  3. How important was this memory problem? Not at all Mildly Moderately Extremely
  4. What did you do to adjust for this problem?
  5. What might you do differently to avoid this kind of problem in the future?

**Time & date completed:**

Asterisk (*) indicates the item was only included in the daily diary completed by the EMMI group.
