# Supplementary material for: Initial Evidence for the Efficacy of an Everyday Memory and Metacognitive Intervention
Source: Innov Aging. 2020 Oct 26;4(6):igaa054. doi: 10.1093/geroni/igaa054 (PMC7729280; doi:10.1093/geroni/igaa054)
Supplement: igaa054_suppl_Supplementary_Materials_S2 [file igaa054_suppl_supplementary_materials_s2.docx]

**Supplementary Materials: SHAPING PHONE CALL BASIC SCRIPT**

| This is a general script designed to guide research team in areas to address. Each phone call, however, is to be tailored to the individual participants and so will vary broadly in content depending on the experiences, needs, and concerns of each participant. The basic ideas of each phone call area to (1) see how they are doing with the intervention components, (2) to see where they might need extra encouragement or assistance, and (3) to provide general support for using the techniques in any way that is suitable.  Suggestions for spoken prose are in italics. | |
| --- | --- |
| *Hello, it’s <insert name> from the EMMI project calling for our check-in. Is now still an okay time to talk?* | |
| *Let’s make sure you have what we need for our review. If you don’t already have it, can you get the daily diary from today? Okay, let’s go through the diary:* | |
| 1. *Did you have any memory challenges today? If yes, what were they?* If no, continue to next item. | |
| 1. *Did you have memory successes? If yes, what were they?* If no, continue to next item. | |
| 1. *Did you use the following techniques today?* | |
| Go through each technique one at a time and for each technique that is endorsed as used, ask these questions:  *What were your challenges with using it?*  *Were you successful?*  *What do you think you could do differently next time?* | |
| For each techniques that wasn’t used, ask these questions:  *What kept you from using the technique (i.e. no need, no opportunity, can’t figure out how to use it in real life, etc)?*  *Let’s think of ways that you might have been able to use the _____ technique ….* | |
| Techniques:   - Active Noticing - Intentional Encoding - Spaced Retrieval - Self-testing | - Mindfulness - External Aids - Implementation intentions - Stop Think Plan Act |
| 1. *Let’s review the goals we set at the last shaping call* (varies by participant). | |
| 1. *Considering everything you’ve shared with us, please rate your use of the trained procedures today on a 1 to 10 scale with 1 being had a lot of difficulty, 5 = some difficulties, and 10 being an excellent use of the procedures). Why did you choose that rating?* | |
| *6) Are there other ways we can be helpful to you today?* | |
